# A novel dual luciferase based high throughput assay to monitor autophagy in real time in yeast *S. cerevisiae*

**DOI:** 10.1016/j.bbrep.2017.07.008

**Published:** 2017-07-20

**Authors:** Piyush Mishra, Shashank Rai, Ravi Manjithaya

**Affiliations:** Autophagy Lab, Molecular Biology and Genetics Unit, Jawaharlal Nehru Centre for Advanced Scientific Research, Jakkur, Bangalore, India

**Keywords:** Autophagy, Luciferase, High throughput screening, Pexophagy, Peroxisome, Small molecules

## Abstract

**Background:**

Macroautophagy is a cellular response to starvation wherein superfluous and damaged cytoplasmic constituents are degraded to provide energy for survival and to maintain cellular homeostasis. Dysfunctional autophagy is attributed to disease progression in several pathological conditions and therefore, autophagy has appeared as a potential pharmacological target for such conditions.

**Objective:**

In search of potential drugs that modulate autophagy, identifying small molecule effectors of autophagy is the primary step. The conventional autophagy assays have a limitation that they cannot be scaled down to a high throughput format, therefore, novel sensitive assays are needed to discover new candidate molecules. Keeping this rationale in mind, a dual luciferase based assay was developed in the yeast *S. cerevisiae* that could measure both selective and general autophagy in real time.

**Methods:**

Firefly and *Renilla* luciferase reporter genes were cloned under POT-1 promoter. Using fatty acid medium the promoter was induced and the luciferase cargo was allowed to build up. The cells were then transferred to starvation conditions to stimulate autophagy and the degradation of luciferase markers was followed with time.

**Results and conclusion:**

The assay was more sensitive than conventional assays and could be scaled down to a 384 well format using an automated system. A good Z-factor score indicated that the assay is highly suitable for High Throughput Screening (HTS) of small molecule libraries. Screening of a small molecule library with our assay identified several known and novel modulators of autophagy.

## Highlights

• A dual luciferase assay for monitoring autophagy was developed that could measure changes in autophagic flux at any step.
• Using the assay, both selective and non-selective forms of autophagy could be measured.
• The assay was more sensitive, robust and kinetic than the conventional autophagy assays.
• Z-factor calculations showed that the assay was highly amenable for a highthroughput setup.
• Screening of small molecule library resulted in identification of several different potential autophagy modulators.

## 1 Introduction

Autophagy, a process of cellular self-cannibalization, is an essential recycling mechanism in eukaryotes. This evolutionarily conserved process involves selective or non-selective degradation of damaged and redundant cellular components by enveloping them inside double membranous structures called autophagosomes and escorting them to the degradation compartments like vacuole (in yeast) or lysosomes (in mammalian cells) The degradation products such as amino acids are recycled back to cytoplasm and utilized in various metabolic pathways [1], [2], [3], [4]. In this way, through constitutive levels of basal autophagy, a cell renews its worn out and unused components to maintain cellular homeostasis [5], [6], [7].

The importance of autophagy for health has been underlined by several reports where dysfunctional autophagy is shown to be implicated in multiple disease conditions [8], [9]. For example, autophagy has been shown to clear smaller protein aggregates inside cell and in its absence these aggregates build up and cause several neurodegenerative disorders [10], [11]. Autophagy also plays critical role in clearing intracellular pathogens like bacteria, virus and hence provide protection from infectious diseases [12], [13], [14], [15]. The role of autophagy is context dependent in cases of cancer. The phrase ‘double edged sword’ has often been ascribed to autophagy for its involvement in cancer [16], [17].

Since the involvement of autophagy in maintaining cellular homeostasis is very important, modulating autophagy levels in pathological situations for therapeutic purposes is of current research interest. Several autophagy modulators have been discovered in the recent past, but very few of them have led to potential candidate drug molecules [18], [19], [20]. There is a lot of scope for discovery of new autophagy modulators that can be later on taken up to clinical trials.

The conventional assays to measure autophagy are qualitative or semi-quantitative in nature [21]. These assays have a limitation that they cannot be scaled down to a high throughput format, which makes the small molecule screening very cumbersome process. Therefore, high throughput screening assays for autophagy is the need of the hour, which can enable us to screen several chemical modulators in a single experiment with all the possible biological and technical replicates. The data obtained from this assay should be in a form which can be directly put to comparison and statistical analysis. Several high throughput assays have been developed to screen for small molecule modulators of autophagy [22], [23]. But these are riddled with some drawbacks. Many of these assays do not directly look at the cargo or possess a higher physiological working range to detect smaller changes in autophagic flux. We circumvented this problem by following the degradation of superfluous autophagic cargo, whose biogenesis could be controlled and therefore, the turnover via autophagy could be measured effectively, which provides a higher range to work with.

Here we present a novel luciferase based high throughput assay for monitoring autophagic flux. The assay is based on measuring the activities of firefly and *Renilla* luciferase, to count the flux of selective and general autophagy respectively using *S. cerevisiae*. The dual luciferase system gives the added advantage of real time, sensitive and kinetic assessment of two different types of autophagy processes simultaneously. The assay was found to be more sensitive and reproducible than the conventional autophagy assays. A very good z-score for the assay indicated that it was amenable to a high throughput setting. After successful automation of the assay, a small molecule library was screened for its effect on the autophagic flux. Several known and novel modulators were identified from the screen. Here we provide data for one such putative modulator of autophagy.

## 2 Materials and methods

### 2.1 Yeast strains and plasmids

Wild type Pot1-GFP strain with genomically tagged GFP to the C terminus of peroxisomal resident protein, Pot1 (HIS selection marker) was obtained from Dr. Rachubinski. Wild type BY4741 and all knockout strains were obtained from **EURO**pean ***S**accharomyces **c**erevisiae* **AR**chive for **F**unctional Analysis (EUROSCARF). *Pichia pastoris* strains (PPY12h) and *S. cerevisiae* shuttle vectors pRS306 (URA) and pRS305 (LEU) were obtained from Prof. Suresh Subramani, UCSD.

### 2.2 Transformation of *S. cerevisiae*

*S. cerevisiae* transformation was done using lithium acetate method. Cells (~10^8^ cells) in early logarithmic phase of growth were harvested and resuspended in transformation mix (final concentrations: 33.3% PEG 3350, 0.1 M lithium acetate, 270 µg/ml salmon sperm DNA, 1–1.5 µg DNA). The cells were then subjected to heat shock at 42 °C for 40 min after which they were harvested and plated onto the selection media plates SD-URA for pRS306P_POT1_-FLUC and SD-LEU for pRS305P_POT1_-RLUC

### 2.3 Pexophagy assay

Pot1-GFP positive strains were allowed to grow till the Absorbance @ 600nm (A_600_) reaches 0.8–1 in YPD. Peroxisome biogenesis was induced by growing these cells in oleate medium (0.1% oleate, 0.5% Tween-40, 0.25% yeast extract, 0.5% peptone, and 5 mM phosphate buffer) for 12 h. Cells were harvested, washed twice to remove traces of media and transferred to starvation medium without nitrogen, at inoculum density A_600_ = 3, to induce pexophagy. Cells were collected at various time intervals after pexophagy induction and processed by TCA method.

### 2.4 TCA precipitation

All samples were collected in 12.5% TCA final concentration and stored at −80 °C for at least half an hour. Later, the samples were thawed on ice and centrifuged for 10 min at 16,000*g*, pellet was washed with 250 µl of ice cold 80% acetone twice and air dried. This pellet was resuspended in 40 µl of 1% SDS- 0.1 N NaOH solution. Sample buffer (5X, 10 µl) was added to the lysate and boiled for 10 min before loading.

### 2.5 Immunoblotting

Total cell lysates were electrophoresed on 12% SDS-PAGE for Pot1-GFP processing pexophagy assay and firefly western blots and transferred onto PVDF membrane at constant current of 2 Ampere for 30 min (Transblot turbo, BIORAD Inc, USA). Transfer was confirmed by Ponceau S staining of blot. Blots were incubated overnight with primary anti-GFP mouse IgG antibody (Roche Diagnostics # 11814460001) in 5% skim milk at 1: 3000 dilution or rabbit anti-Firefly antibody (Abcam # ab21176) at 1:3000 dilution. Secondary antibody used at 1:10,000 was goat anti-mouse (Biorad # 172-1011) or goat anti- rabbit antibody (Biorad # 172-1019) conjugated to HRP. Blots were developed by using ECL substrate (Thermo Scientific # 34087) and images captured using auto capture program in Syngene G-Box, UK. Image J (NIH) was used for quantitation of band intensities.

### 2.6 Fluorescence microscopy

Pot1-GFP labeled cells growing in mid log phase were transferred to oleate medium. These cells were washed and transferred to starvation medium and split into two batches. Cells were collected after every 30 min and mounted on 2% agarose pad and visualized using Delta vision microscope Olympus 60X/1.42, Plan ApoN.

### 2.7 Immunofluorescence

Cells were grown in YPD to A_600_ = 0.6–0.8 and transferred to oleate medium for induction of peroxisome biogenesis at A_600_ = 1. Ten milliliters culture of cells was harvested after overnight incubation in oleate by centrifugation at room temperature and resuspended in 5 ml of freshly prepared 2X fixative (50 mM potassium phosphate buffer, pH 6.5, 1 mM MgCl_2_, 4% formaldehyde). Cells were fixed for 2 h at room temperature in a 15-ml tube with end to end mixing. Cells were collected by centrifugation for 3 min at 1000*g* and resuspended in 5 ml of freshly prepared wash buffer (100 mM, potassium phosphate buffer, pH 7.5, 1 mM MgCl_2_) and centrifuged again as above. Cells were resuspended in wash buffer to an A_600_ of 10 and 0.6 µl of 2-mercaptoethanol and 20 µl of 10 mg/ml Zymolyase 20 T were added to 100 µl of cell suspension. Cell suspension was incubated at room temperature for 15–30 min with mixing end-over-end for spheroplasting. Spheroplasts were centrifuged for 2 min at 400*g* and resuspended in 100 µl of wash buffer and centrifuged again. Final resuspension was done in 100 µl of wash buffer. The glass slide was charged with 0.1% polylysine (Sigma) and 20 µl of spheroplasts was added to each well. Spheroplasts were post-fixed by immersing the slide glass in acetone precooled to −20 °C for 5 min at −20 °C. Blocking was done using a drop of PBS-Block (PBS, pH 7.4, 0.1% BSA, 1% skim milk) for 30 min. Cells were incubated with primary antibody mixture in PBS block (Rabbit anti-firefly luciferase from Abcam # ab21176; Mouse anti-*Renilla* luciferase from Millipore # MAB4400) post blocking and incubated overnight at 4 °C in a humid chamber. Slide was washed with PBS block several times and incubated in secondary antibody (Anti-rabbit Atto-550, Sigma # 43328 and Anti-mouse Atto-550, Sigma # 43394) mixture in PBS block and incubated at room temperature in dark-humid chamber for 1–2 h. Mounting medium (Vectashield without DAPI # H-1000) was added and the slide was sealed and observed under fluorescence microscope from Zeiss.

### 2.8 Luciferase assay

Cells were grown in YPD and transferred to oleate medium for peroxisome biogenesis and incubated overnight at 30 °C on a shaker at 250 rpm. Cells were then changed to starvation medium to induce pexophagy (SD-N, 0.17% YNB without ammonium sulphate and 2% glucose). Samples (A_600_ = 3 equivalent) were processed at the mentioned time-points using passive lysis buffer (Promega Dual Luciferase Reporter assay system # E1910). Firefly luciferase followed by *Renilla* luciferase activities were measured after adding their respective substrates in the samples.

## 3 Results

### 3.1 Development of dual luciferase assay for measuring autophagic flux

The principle of the assay involves simultaneously building up the levels of firefly luciferase and *Renilla* luciferase during peroxisome biogenesis and then following the degradation of the luciferase activities over time, upon induction of autophagy. To achieve this, firefly and *Renilla* luciferase expressions were driven by the POT1 (Peroxisomal Thiolase-1) promoter which was activated during peroxisome biogenesis [24]. Firefly luciferase contains an N-terminal peroxisomal targeting signal ‘PTS-1’ (SKL) [25] that escorted it to peroxisomal membrane. *Renilla* luciferase, on the other hand, was cytosolic. The rate of autophagic cargo decay, upon induction of autophagy, was reflected in the decrease in firefly luciferase (targeted to the peroxisomes) and *Renilla* luciferase values. Decrease in firefly luciferase gave a read out for selective degradation of peroxisomes (a selective form of autophagy) [26], [27], [28] whereas *Renilla* luciferase degradation represented rate of general (non-selective) autophagy.

**Fig. 1:**
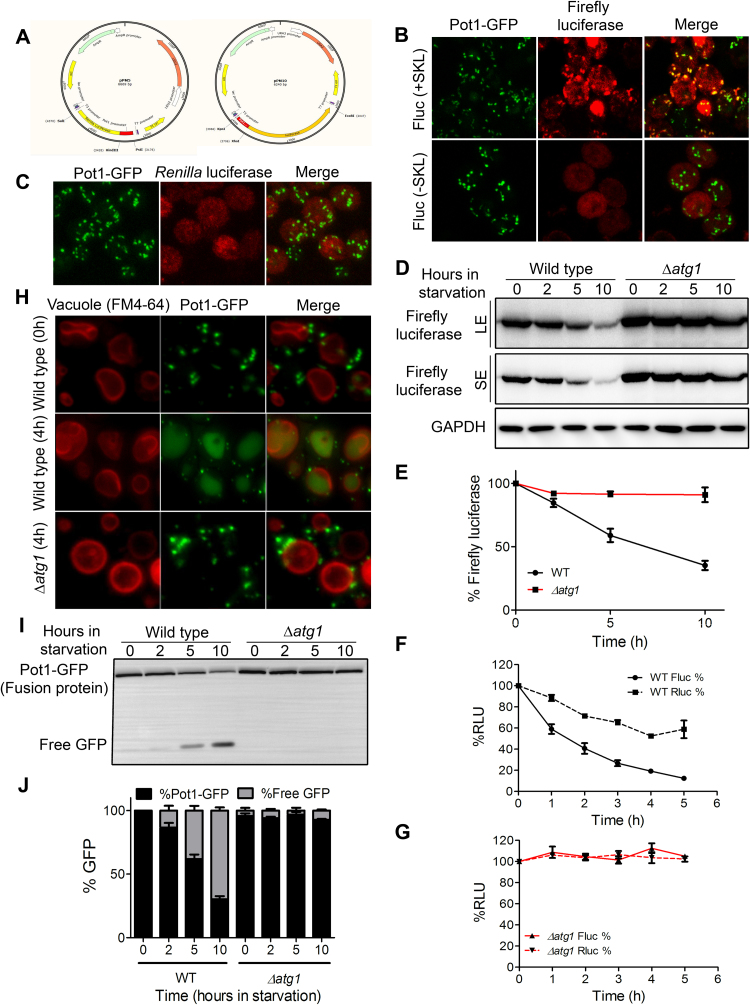
**Dual luciferase assay for monitoring autophagy in budding yeast.** A) Shuttle vectors pPM5 and pPM10 were designed with *Renilla* luciferase and firefly luciferase gene respectively under the fatty acid driven promoter for Peroxisomal thiolase gene (POT1). *Renilla* luciferase was cloned without any signaling sequence whereas firefly luciferase was tagged with three amino acid long Peroxisomal Targetting sequence (PTS-1), SKL at its N-terminal. This directs the firefly luciferase gene to the peroxisomes. The principle of the assay involves simultaneously turning on the expression of firefly and *Renilla* luciferase during peroxisome biogenesis and then following their degradation via autophagy under starvation conditions. B) Fluorescence microscopy showing localization of firefly luciferase with the peroxisomal resident protein (Pot1-GFP). Firefly luciferase with N-terminal signal peptide colocalized with the peroxisomal marker whereas firefly luciferase without the signal peptide remained cytosolic. C) Immunolocalization of *Renilla* luciferase in the cytosol. D) Degradation of firefly luciferase protein under autophagy inducing conditions (nitrogen starvation) in wild type and autophagy mutant (Δ*atg1*) cells. E) Quantification of decay in firefly luciferase levels showing the degradation is autophagy dependent. F) and G) Dual luciferase assay for monitoring autophagy in wild type and Δ*atg1* strains respectively using firefly and *Renilla* luciferase as markers for following rates of selective and general autophagy. H) Conventional autophagy assays for degradation of peroxisomes in wild type and autophagy mutant using fluorescence microscopy. Wild type cells when moved to starvation conditions led to degradation of peroxisomes, shown here with the diffused GFP signal inside the vacuole. Autophagy mutant strain on the other hand did not show any diffused GFP inside the vacuole and intact peroxisomes were observed in the cytosol. I) Immunoblotting showing degradation of peroxisomal protein Pot1-GFP through autophagy. Free-GFP was observed in wild type cells but not in autophagy mutant where only the fusion protein was observed. J) Quantification of Immunoblot for Pot1-GFP processing assay (pexophagy assay).

The *S. cerevisiae* shuttle vectors pRS306 (URA) and pRS305 (LEU) were used to clone the POT1 promoter with the firefly and *Renilla* luciferase genes respectively. The fatty acid responsive region of the POT1 promoter was amplified from yeast genomic DNA and along with the firefly and *Renilla* luciferase genes (from commonly available sources), was cloned into these vectors to obtain the constructs pPM10 and pPM5 respectively (Fig. 1A). These plasmid constructs were linearized using suitable restriction enzymes within the selection markers and transformed into wild type strains of *S. cerevisiae* for genomic integration. Similarly, several autophagy mutant strains such as *Δatg1,* Δ*atg5* and Δ*pep4* were co-transformed with firefly and *Renilla* luciferase vectors. Firefly luciferase with the N-terminal PTS-1 signal sequence co-localized with the peroxisomal resident protein Pot-1 tagged with GFP whereas firefly luciferase lacking this signal sequence was cytosolic (Fig. 1B). *Renilla* luciferase also localized to the cytosol (Fig. 1C).

Immunoblot analysis for the firefly luciferase protein levels showed an autophagy dependent degradation (Fig. 1D and 1E). Wild type cells showed decrease in the levels of firefly luciferase on autophagy induction. Core autophagy mutant Δ*atg1* on the other hand did not show any decrease in firefly luciferase levels.

### 3.2 Luciferase assay versus conventional assays

The dual luciferase positive strains were screened and grown in fatty acid containing medium to induce the biogenesis of peroxisomes. The firefly and *Renilla* luciferase, being under fatty acid responsive promoter, were expressed under these conditions. After 12 h of induction, the cells were transferred to nitrogen starvation medium to stimulate autophagy. The degradation of luciferase markers was followed, with time, as readout for autophagic flux. Decrease in levels of firefly luciferase, which was targeted to peroxisomes, indicated selective degradation of peroxisomes through autophagy (pexophagy). Rate of degradation of *Renilla* luciferase indicated random degradation of cytoplasmic contents via non-selective autophagy (Fig. 1F). Wild type cells showed decay in luciferase markers with time (Fig. 1F) whereas autophagy mutant (Δ*atg1*) did not show any degradation, showing that the breakdown of luciferase markers is exclusively autophagy dependent (Fig. 1G).

To validate the luciferase assay developed in the laboratory, it was compared to the conventional immunoblotting and fluorescence microscopy based autophagy assays using degradation of peroxisomal marker as the readout (Pot1-GFP processing assay). In the luciferase assay (Fig. 1F and 1G), the enzymatic activity in the wild type cells decreased over time whereas the autophagy mutant showed no decrease in the activity upon autophagy induction. Fluorescence microscopy with the wild type and autophagy mutant also gave a similar trend. Wild type cells when moved to starvation conditions led to degradation of peroxisomes shown here with the diffused GFP signal inside the vacuole. Autophagy mutant strain on the other hand did not show any diffused GFP inside the vacuole and intact peroxisomes were observed in the cytosol (Fig. 1H). This was also consistent with the immunoblotting for Pot1-GFP processing assay (Fig. 1I and 1J), wherein the wild type cells showed degradation of peroxisomal protein, observed as decrease in Pot1-GFP levels and appearance of free GFP. Autophagy mutant on the other hand did not show any degradation of autophagic cargo.

More importantly, when the levels of firefly activity were compared with conventional assays like Pot1-GFP processing assay, the luciferase assay was found to be more sensitive. In the wild type situation, the firefly luciferase activity decreased to less than 50% within 2 h (Fig. 1F and 1G), whereas the 50% decrease could be detected with the help of Pot1-GFP levels only after 6 h (Fig. 1I and 1J). This indicated that smaller changes in the cargo flux can be detected better using the luciferase reporter than the conventional assays. Since, the luciferase assay could be adapted to a multi well plate format and for shorter time durations, it is highly amenable for high throughput studies for the screening of small molecule modulators of autophagy.

### 3.3 Assay automation

**Fig. 2:**
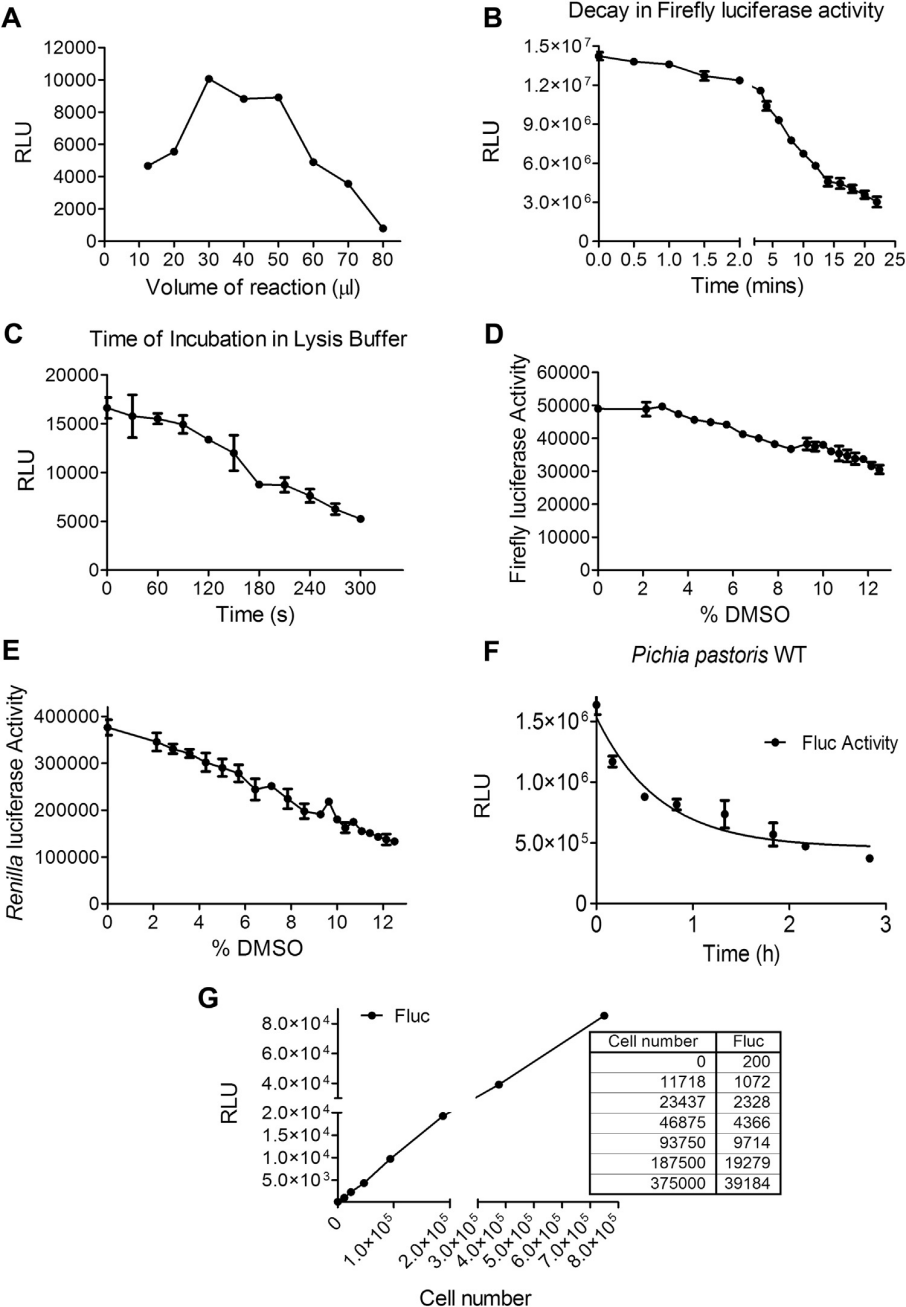
**Optimization of luciferase assay for a high throughput setting.** A) Miniaturization of luciferase assay in a 384 well format. Optimal volume of reaction was obtained with the maximum luciferase activity. B) The stability of firefly luciferase activity under the reaction conditions was determined. This provided the window period over which the firefly luciferase reading could be measured without any loss of signal. C) Time taken for lysis of cells in a 384 well plate using the Passive Lysis Buffer. D) Effect of DMSO concentration on firefly luciferase activity and E) *Renilla* luciferase activity. F) Firefly luciferase assay was also done in *Pichia pastoris* wild type cells showing a similar trend and the rate of degradation as *Saccharomyces cerevisiae*. G) Change in firefly luciferase activity with increasing number of cells.

In order to perform the dual luciferase assay, the Dual Luciferase Reporter assay kit from Promega was used. First, the optimum volume to carry out the assay in a 96 or 384 well format was determined (Fig. 2A). A volume of 40 µl with an incubation time of 90 s was finalized. The assay was further standardized for the stability of the substrate and the enzyme activity over time (Fig. 2B and 2C). The dual luciferase assay was performed by lysing the cells first using the passive lysis buffer, followed by readout for firefly luciferase by adding its substrate. The substrate for *Renilla* luciferase was then added which also acted as a quencher for firefly luciferase activity so that only the *Renilla* luciferase activity could be measured.

Since most of the drugs in a library are dissolved in DMSO, the effect of DMSO itself on the assay or luciferase activities was tested. DMSO concentrations from 0% to 12% were used. Assay was done up to 3 h in a 96 well plate. It was seen that a concentration of up to 4% DMSO did not substantially affect firefly luciferase activity (Fig. 2D) whereas a concentration till 2% DMSO had no effect on *Renilla* luciferase activity (Fig. 2E). Firefly luciferase activity provided more robustness, tolerance to DMSO and a broader range to monitor the flux than *Renilla* luciferase. Therefore, for carrying out the small molecule screen, firefly luciferase activity was chosen over *Renilla* luciferase as the readout. Firefly luciferase assay was also standardized for another yeast system *Pichia pastoris,* that also showed a similar trend in decay of luciferase activity under autophagy conditions as shown earlier in *Saccharomyces cerevisiae* wild type cells (Fig. 2F).

Finally the linear range for the luciferase activity with respect to cell number was determined (Fig. 2G). The luciferase activity and the change in cell number also followed a linear correlation over a wide range.

### 3.4 Dual luciferase assay in a high throughput setting

**Fig. 3:**
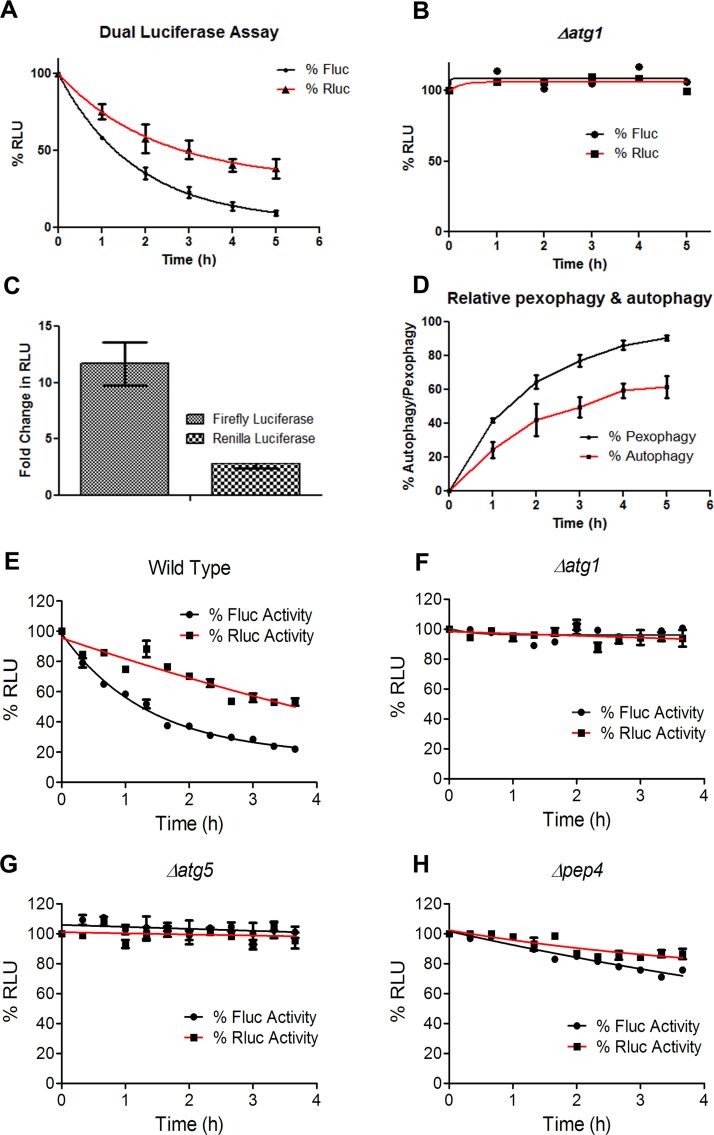
**Dual luciferase assay in a high throughput format.** A) Dual luciferase assay done in 96 well plate in wild type cells and B) autophagy mutant (Δ*atg1*). C) Fold change in the luciferase activity over the duration of the assay was calculated for wild type cells by taking the ratio of initial reading at 0 h of starvation by final reading at 5 h. Firefly luciferase showed more change in its activity than *Renilla* luciferase. D) Decay in firefly luciferase activity represented pexophagy (degradation of peroxisomes through selective autophagy) whereas *Renilla* luciferase activity showed non-selective autophagy. E) Dual luciferase assay done in a 384 well format for wild type cells and autophagy mutants F) Δ*atg1*, G) Δ*atg5* and H) Δ*pep4.*

After several rounds of standardizations, the assay was carried out in a 96 well format. The pattern of degradation of luciferase activities was similar to that at the flask level for wild type cells and Δ*atg1* cells (Fig. 3A and 3B). However, it was observed that the decrease in firefly activity was much faster than the *Renilla* luciferase activity (Fig. 3C), suggesting that following a cargo that is destined for capture and degradation is a better substrate than the cytosolic cargo, where only a part of it is taken up for degradation. Pexophagy rates as determined by decay in firefly luciferase activity were more as compared to non-selective form of autophagy shown by *Renilla* luciferase levels (Fig. 3D).

After successfully doing the manual assay in 96 well format, full automation of the assay steps was carried out in a multiplate reader (Varioskan Flash, Thermo Scientific and FLUOstar Omega from BMG Labtech) in a 384 well plate. The results obtained were in concordance with what was seen with manual assay at flask level or the 96 well plate level (Fig. 3E and 3F). The firefly and *Renilla* luciferase activities showed a considerable amount of decrease (more in case of firefly luciferase) in the wild type cells when transferred to starvation conditions. However, the activities remained constant with time in case of autophagy mutants blocked at different steps of autophagic degradation: Δ*atg1*, Δ*atg5* and Δ*pep4* (Fig. 3E-3H).

### 3.5 Z-factor calculation

The Z-factor is a measure of the quality of a high throughput screening (HTS) system. The Z-factor predicts if useful data could be expected if the assay were scaled up to millions of samples. Z-factor was calculated for 5 independent assays done in triplicates in 384 well format, for both firefly as well as *Renilla* luciferase activities. It was found that the values obtained were greater than 0.8 for both the luciferases (Z-factor for firefly luciferase = 0.8628 ± 0.03481; Z-factor for *Renilla* luciferase = 0.8224 ± 0.03879), which suggested that our assay is very robust, reproducible and when scaled up to millions of compounds would give very less false positives and better reliability.

### 3.6 Screening of compounds and identification of hits

**Fig. 4:**
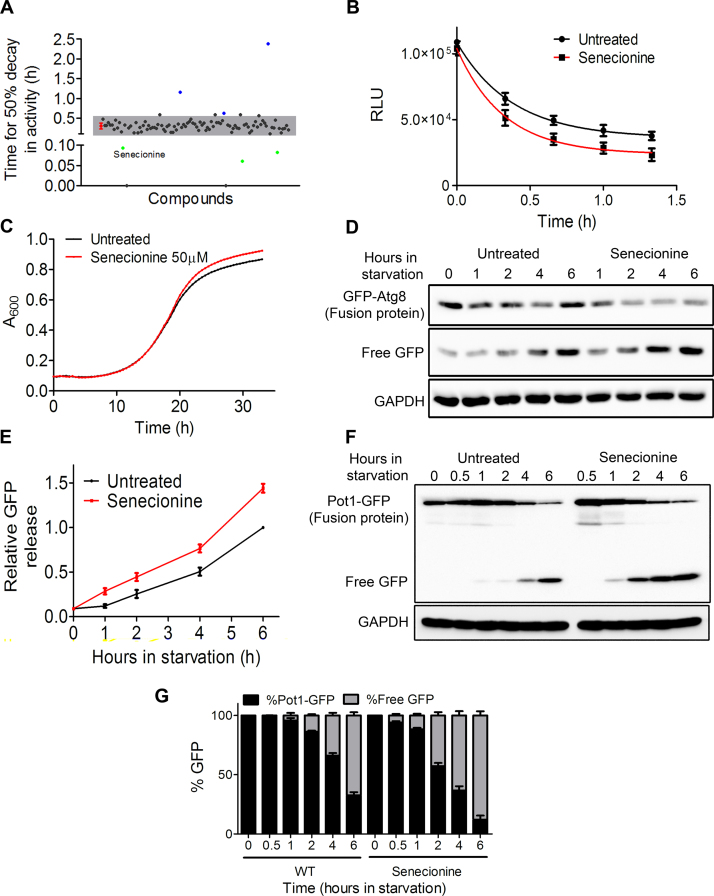
**Small molecule screening and identification of hits.** A) Graph representing the screening of Enzo library of 502 natural compounds. Red dot represents the control cells without any compound treatment with its error bars depicting the standard deviation (SD). The grey shaded region represents 3 SD area. Individual black dots represent each compound from the library. Any compound that affected the time of decay in firefly luciferase activity by more than 3 SD units from the control was considered as a hit. Any dots lying outside the shaded region of 3 SD units represent a putative hit from the primary screening. The green dots represent the putative enhancers whereas blue dots represent putative inhibitors of autophagy. B) Luciferase assay in 5 replicates for a putative autophagy enhancer ‘Senecionine’ obtained from the screen. C) Growth curve showing that Senecionine at 50 µM did not affect the growth of cells. D) Validation of the hit using secondary autophagy assays. General autophagy assay using GFP-Atg8 fusion protein as the marker was carried out. Autophagy is represented by release of free GFP. E) Densitometric analysis of free GFP band revealed that Senecionine increased the levels of free GFP as compared to untreated control. F) Conventional pexophagy assay for showing selective form of autophagy using peroxisomal resident protein Pot1, fused with GFP as a marker. Treatment with Senecionine increased the autophagic degradation of peroxisomes shown by more decrease in the fusion protein and release of free GFP at an earlier time point as compared to the untreated control as also confirmed by G) densitometric analysis of fusion protein and free GFP form. Figure legend needs justification.

The library of 502 natural compounds from Enzo was tested for its effect on autophagy using the luciferase based HTS assay. The rates of degradation of luciferase cargo in the untreated cells were compared to the ones treated with 50 µM concentration of the compounds. The time taken for 50% decrease in cargo activity was taken as the criteria for comparing the control with the compounds. The compounds that differed from the control in change of luciferase activity by 3 SD (Standard Deviation) units were considered significant (Fig. 4A).

Several hits were obtained from the primary screen. Many known autophagy modulators were also reflected as hits along with some novel molecules (Table 1). The hits were further validated using the secondary assays like immunoblotting and microscopy based assays.

**Table 1:** List of known autophagy modulators obtained from screening of Enzo library.

**Enhancers**	**Inhibitors**
Rapamycin	Etoposide
CAPE	Colchicine
Grayanotoxin III	Gossypol
Vitexin	Cinobufagin
Neomycin	Cryptotanshinone
Retinoic acid	Wortmannin
Rottlerin	Brefeldin A
Curcumin	Cyclohexamide
Doxorubicin	E-64
Caffeine	Taxol
	Vinblastine sulphate

Composite list of all the known autophagy modulators (hits) obtained from the screening of the Enzo library using the luciferase based assay. The screen identified both enhancers and inhibitors of autophagy.

One of the putative hits, Senecionine was further characterized using secondary assays in yeast. Luciferase assay in 5 replicates for a putative autophagy enhancer ‘Senecionine’ obtained from the screen, showed a decrease of firefly luciferase activity more than the untreated control (Fig. 4B). Growth curve for Senecionine at 50 µM showed that the compound was not toxic and did not affect the growth of cells at this concentration (Fig. 4C). Validation of the hit using secondary autophagy assays was carried out. General autophagy assay using GFP-Atg8 fusion protein as the marker where autophagy was represented by release of free GFP was performed. Seneconine showed more accumulation in free GFP and decrease in the fusion protein GFP-Atg8 as compared to the control (Fig. 4D). Densitometric analysis of free GFP release also showed Senecionine as a potent autophagy enhancer (Fig. 4E). Conventional pexophagy assay for showing selective form of autophagy, using peroxisomal resident protein Pot1, C-terminally Pot1, C-terminally tagged with GFP as a marker was also carried out. Treatment with Senecionine increased the autophagic degradation of peroxisomes shown by more decrease in the fusion protein and release of free GFP at an earlier time point as compared to the untreated control (Fig. 4F and 4G). These assays validated the compound to be a potent enhancer of autophagy.

## 4 Discussion

Basal levels of autophagy take place in all the cells in order to maintain cellular homeostasis [7]. However, in several diseases the process of autophagy is perturbed [9]. Therefore, autophagy has emerged as an attractive target for the treatment of various disease conditions in the recent years. Studies have shown that modulating autophagy has positive outcomes in the diseases such as diabetes, cancers [29], [30], [31], neurodegenerative disorders [32], [33] and some infectious diseases [12], [15]. Modulating autophagic activity has resulted in increased killing of intracellular mycobacteria [12]. Pharmacologically, small molecules targeting autophagy have been shown to be effective in clearing protein aggregates in a Huntington model system [18], [33], [34]. It has been proposed that pharmacological intervention of the autophagy process can lead to better understanding of various degenerative disorders and cancers. In view of this, identification of new small molecule modulators of autophagy is the first step.

In the past, many drug screens using several different autophagy readouts have been undertaken to find out new drug candidates, that affect autophagy using yeast or mammalian cells as models, resulting in drugs of potential clinical utility [33], [35], [36], [37]. These assays, although quantitative, lack one of several important parameters such as build-up of autophagic flux, sensitivity, ease of handling, broader range to work with, and autophagy readout in live cells in real time. An ideal assay would incorporate all these properties in a single high throughput format. In this study we introduce a novel luciferase based assay to monitor autophagy in real time that fulfills all these criteria. Unlike the cytoplasmic autophagic flux of proteins, degradation of an organelle like peroxisomes is a better alternative to the pre-existing assays since these organelles can build up in number and bulk degradation, along with its intra-organelle components, occurs in a relatively short period. This provides a higher range of autophagic cargo decay to work with, resulting in substantial increase in the dynamic range of the assay. Calculation of statistical parameters such as Z-factor showed that the assay is highly suitable for small molecule screening and that the assay would be useful in a high throughput setting. Screening of small molecule library using the assay yielded several known as well as novel autophagy modulators, further highlighting the effectiveness of the assay. One of the putative autophagy enhancers; Senecionine has been validated in this study. Senecionine is a plant alkaloid obtained from herbs of *Senecio* species. *Senecio* herb is used as a folk remedy for diabetes mellitus, hemorrhage, high blood pressure, for spasms, and as a uterine stimulant. However, no molecular target for the compound is known or reported in the available literature. Thus, identifying the target of this molecule would potentially reveal mechanism of autophagy modulation. In addition, as the assay is not directed towards a particular target, it could detect modulators that affect any step of autophagic flux from biogenesis to cargo degradation.

Hence, our luciferase based pexophagy assay provides the convenience of performing a small molecule high throughput screening, using yeast as the model system. Owing to the conserved nature of autophagy, the hits can be further tested in higher eukaryotes and the leads can be tested in various autophagy dependent disease models, which will provide a new approach for discovering molecules that affect host pathogen interaction and also in case of neurodegenerative disorders and cancer disease models.

## Disclosure of potential conflicts of interest

The authors declare no potential conflict of interest.
